# The Maxillofacial Service of the American Army in the War*Read before the Section on Surgery, General and Abdominal, at the Seventieth Annual Session of the American Medical Association, Atlantic City, N. J., June. 1919. Reprinted from the *Journal A. M. A.*, Aug. 2, 1919.

**Published:** 1919-08

**Authors:** V. P. Blair


### THE MAXILLOFACIAL SERVICE OF THE AMERICAN ARMY IN THE WAR *

BY V. P. BLAIR, M.D.

Among the many special problems listed on the preparation program of the Surgeon-General of the Army was the care of injuries of the face and jaws. These had been a particularly trying problem in the Allied armies in the earlier part of the war. In July, 1917, a section of the Division of Surgery of the Head was organized to take over this duty. The motif of preparation was the conviction that the earlier proper treatment could be instituted and continuously carried out, to a proportionate extent would conservation displace reconstruction. To make this practicable would require, first, a sufficiently large personnel to be available at every advanced and at all intermediate and base hospitals; secondly, a definite general

* Read before the Section on Surgery, General and Abdominal, at the Seventieth Annual Session of the American Medical Association, Atlantic City, N. J., June, 1919. Reprinted from the *Journal A. M. A.*, Aug. 2, 1919.

plan of treatment which, instituted in the advanced hospitals, would be carried out without radical change in each of the hospitals to which the wounded would be subsequently evacuated, and, thirdly, and not unimportant, suitable equipment.

The proper treatment of maxillofacial injuries rests on the same surgical principles as that of wounds in any other part of the body; but because the proper splinting of a fractured jaw requires dental splints, or splints with dental attachment, and because few surgeons have familiarized themselves with the physiology and the special pathology of the oral structures, to do the work efficiently requires, as a rule, the co-operation of a surgeon with a dental surgeon who has made a special study of the subject.

We had available in this country a large number of dental surgeons who had made this special study, and of surgeons fitted for this co-operation.

### EARLY PREPARATION

The Surgeon-General, with the generous and whole-hearted co-operation of both the medical and dental professions, and especially of the institutions to be named, established special short courses in the Northwestern University Dental School, in the Evans Dental Institute, University of Pennsylvania, and in Washington University; but the selection of teachers was not confined to the faculties of these particular schools. A large number of surgeons and dentists were sent to take the courses, not with the idea that they could be created into specialists, but that the dentists and surgeons could be shown the plan of co-operation, while splint-making, the more likely problems, and the anatomy of the parts were being reviewed. Not the least important of the things accomplished by these schools was the data obtained as to the qualifications of officers for the work, which requires also the co-operation of the ophthalmologist and otorhinologist; and the close association of the latter sections with the maxillofacial in the Division of Surgery of the Head simplified the arrangement for this co-operation, which has been harmoniously carried out ever since.

In the matter of equipment, everything asked for was allowed by the Surgeon-General and assembled into sets adapted to advanced and base hospitals.

### FRENCH AND BRITISH CO-OPERATION

In April, 1918, the preliminary work in this country having been carried as far as practicable, the Surgeon-General sent a unit of about twenty surgeons and twenty dental surgeons to the chief surgeon of the American Expeditionary Forces with the request that they be given the opportunity to observe the method in practice for caring for face and jaw injuries in the hospitals of our allies, and through the courtesy of the authorities they were distributed among several British and French hospitals especially devoted to this work, namely, the various Paris hospitals, Queen's Hospital at Sidcup, King George Hospital, England, and British General Hospital No. 20 at Boulogne. Later, as their services were required in our own hospitals, they were gradually withdrawn from these stations. It would be very ungracious if I did not pause in this hurried review to acknowledge the debt we owe to the British and French authorities for the help they gave us in this manner, and I mention, among others, especially Collyer, Gilles, Cole and their staffs; Lemaitre, Pont, the late Moristan, Villain and Kazanjian, who did everything they could to make their experiences available to us.

### ORGANIZATION OF OPERATIONS IN FRANCE

Shortly after we arrived in France, a senior consultant for maxillofacial surgery for the American Expeditionary Forces was appointed, and a policy for the care of these cases was outlined, which differed a little from the plan contemplated by the Surgeon-General. Information as to the general plan for evacuation and treatment of these patients was sent out. France was divided into seven areas, to each of which a local or area consultant for maxillofacial surgery was appointed. In each American hospital center the cases were concentrated as far as practicable in one hospital, where they were attended by a local consultant for the center, in co-operation with specially selected dental surgeons. In each area, comprising several hospital centers, the area consultant, while he was free to travel throughout his area, was attached to a certain hospital to which he could request to have evacuated any patient who could best be treated at his own hospital, or to Hospital No. 115 at Vichy. The latter had been especially designated and equipped to care for face and head injuries.

### LACK OF SURGEONS AND EQUIPMENT

The atmosphere of war, in spite of intense concentration, is one of distraction. All plans of specialization are more or less ideal, and it takes time to put any subordinate plan into operation, especially if it is far-reaching in its application. Each hospital organized in the Surgeon-General's office had attached to it a surgeon and a dental surgeon designated for this work; but in the earlier part of the severe fighting, all specialization was overshadowed by more vital problems. It is no secret that, owing to the lack of transportation, we were short of surgeons and dental surgeons in the American Expeditionary Forces, and that at Chateau-Thierry most of the surgical consultants were ordered to form general operating teams, and they stood twelve-hour shifts at the table at any hospital where they could find space; while the dental surgeons were often giving anesthetics, carrying the stretchers or giving first aid treatment. I have seen a hospital with an operating room capacity of 360 cases per twenty-four hours, at 2 o'clock in the afternoon, in all the confusion of raising the tents, and which, before seven the next morning had received 750 severely wounded—with the stream unabated; and I know of instances of even greater congestion. At one rail head, two consultants formed a team and carried stretchers for nearly a day.

The foregoing instances are mentioned to illustrate just one of the reasons why it took time to establish these special services.

I believe our service abroad had some success in its work. In my own mind I am very enthusiastic about its accomplishments. When all of the special records of these cases are received from abroad, we shall have definite figures to judge from. Until then, its accomplishments can be measured only by inference. After August 20, there was a breathing spell, during which this service really got on its feet; and from the battle of the Argonne, September 25, it was on the job and delivering the goods at all stations, if I may be allowed to use this expression. It was a bit of a handicap that little of our special equipment ever became available; but shells and food take precedence over other things. This worried the dental surgeons, who were not backward in worrying the consultant, and the consultant worried every one.

During the St. Mihiel drive, a dental surgeon of a mobile hospital telephoned the local consultant of the advanced area, asking what he could use to take care of the jaw injuries that were accumulating. The consultant replied, "Use your wits," and slammed down the telephone. He had been asked this question once too often. To the credit of our dental surgeons, they used their wits, and by piecing out our own supplies with what the chief dental surgeon could obtain from the French; by beating two-franc pieces into splints; by robbing the shell-cut telephone wires, and by cutting meat tins into splints, from the beginning of the Argonne there were few patients who reached the base hospitals unsplinted.

### NUMBER AND RECORD OF CASES

We have at present no definite figures as to the total number of these cases. Taking the statistics for the Union forces in the Civil War, the Prussian forces for the War of 1870-1871, and the British forces for the present war up to Christmas, 1917, as a basis for calculation, they should average at least 1 per cent. of all wounds, which, according to the last report of wounded May 31, 1919, would give a total of more than 2,371 cases.

There have been evacuated to this country to date about 600 patients, and practically no more remain abroad. Of these 600, 260 have been discharged, and eighty-five are ready to be discharged. For the 339 patients now in hospitals, making liberal estimates of the time necessary for total recovery, there are ninety-two who will require two months; eighty-two, four months; fifty, six months; eleven, eight months; seven, twelve months, and one, eighteen months, before they can be discharged as well; but it is estimated that the time that will necessarily be spent in the hospital will average from two weeks to ninety days for the foregoing cases. In my recent inspection of four centers I did not see one case in which there was a free discharge of pus; in fact, any pus at all in these cases is very rare.

In this country, these cases have been concentrated in four hospitals, and an arrangement with the Public Health Service is under way which will assure the continuance of their treatment nearer their homes of men who wish their discharge.

In the general planning, the matter of records was included. Abroad, every maxillofacial case was recorded in some detail on a special form designed for that purpose, and these are to be returned to this country, to be correlated with the records here. At certain centers abroad, photographs, casts and wax models were made of the earlier conditions, and tracings of the roentgenograms. At each of the four centers in this country there is attached to this service on artist, a sculptor, a photographer and a stenographer, and an officer is assigned to supervise and assemble the records. The Surgeon-General has sent out instructions as to the general plan of these records, and has ordered that on completion of the work all should be sent to Washington to be assembled with the records from abroad, for a permanent exhibit in the Army Medical School Museum. Part of this record is on exhibit in the Exhibition Hall here.

### MASKS

The matter of making masks to camouflage these face injuries was investigated extensively by the Surgeon-General, and a personnel trained for the work. A few were made of our soldiers by the Red Cross in Paris, and several here, by our own staff; but the men will not wear them, preferring plastic reconstruction. There is not, to my knowledge at present, a single mask in use by our soldiers, and Major Gilles of the British service tells me that the soldiers have the same preference. We do, however, use prosthetic reconstructions within the mouth.

### IMPORTANCE OF EARLY TREATMENT

In this review of the work it is impossible even to mention technic, but there is one thing that observation has strongly emphasized, and that is that delayed recovery and restoration are on the whole much more dependent on delayed treatment and infection than on primary loss of tissue, and that early treatment will prevent or control infection. It was brought out more strongly than ever before that infection is the chief factor in delayed union or reformation of the jaw bones, not necessarily accompanied by free flowing pus or extensive induration, but a quiet process, often difficult to detect, usually kept up by want of proper drainage and the presence of a foreign body, most likely on exposed tooth root or a piece of denuded bone. No matter how often the observation is repeated, it is interesting to note with what rapidity bony consolidation frequently follows the removal of a tooth or bone spicula from or near a fracture line that was apparently perfectly clean, but in which union had hung fire for months.

The great majority of cases requiring bone-grafting do so not from the primary loss of all bone-forming tissue, but because of one or several of the following reasons: The bone-forming structures are destroyed by infection, which causes scar tissue to replace bone reproduction; or there has been a want of proper early splinting, which in itself predisposes to infection. It is usually a combination of both these factors that is responsible for a total loss of a segment, which is more apt to occur if, at the time of the injury, bone fragments are removed. Instances in which all of the bone-forming tissues are destroyed by the original injury are the exception. Altogether, about seventy-five cases will require grafting, or have been grafted, for bone loss in the lower jaw. Most of these will be pedicle grafts, which require less time for union and are more certain in result than the free bone graft. It has been the policy throughout to restore the normal occlusions rather than to bridge defects by shortening the body of the jaw.

The last jaw case in which I saw an operation performed in the American Expeditionary Forces was a fair illustration of the working of our plan for conservation. Going into the operating tent of a mobile hospital, I watched a young surgeon whom I did not know and one of our dental surgeons assemble, splint and repair a mandible that was shattered from angle to angle, with the lip, chin and floor of the mouth torn open to the hyoid. When they completed their work, the bony fragments were in their proper position, and the soft parts repaired, all with proper drainage. There was left only a slight defect in the lower lip. Had this treatment been delayed thirty-six hours, the patient would have represented one of those horrible cases of total loss of the chin and body of the mandible which have been so frequently depicted. I never saw the patient again, that is, to recognize him, but with the subsequent treatment we were prepared to give it is probable that the soft tissues healed primarily with bony consolidation within three to six months.

NECESSITY OF DENTAL SURGERY

There are two other cases coming to my earliest observation, between which I have frequently made mental comparison. Before co-operation had been made practicable, April 25, 1918, one of the best operators in the American Expeditionary Forces had treated a case with bad laceration and comminution by ordinary surgical methods, with wiring of the fragments. The patient was evacuated and subsequently treated as though his were a general surgical case. Within a short period there entered the service of the same surgeon a rather similar case, which was treated by him and the dental surgeon jointly. I re-encountered the second patient a month later at a base hospital. The soft tissues had healed with linear scars, the jaw fragments were firmly splinted and in good position, and there was no pus. Six months later I again found the first patient; he was profoundly septic, with fragments of dead bone protruding from the cheek wounds, and there was great deformity. He still will require a bone graft on each side of the body of the lower jaw, and has no lower teeth by which to fix the fragments. "One swallow does not make the summer," but one case will illustrate the surgical principle.

